# GHK Peptide as a Natural Modulator of Multiple Cellular Pathways in Skin Regeneration

**DOI:** 10.1155/2015/648108

**Published:** 2015-07-07

**Authors:** Loren Pickart, Jessica Michelle Vasquez-Soltero, Anna Margolina

**Affiliations:** Skin Biology, Research & Development Department, 4122 Factoria Boulevard, SE Suite No. 200 Bellevue, WA 98006, USA

## Abstract

GHK (glycyl-L-histidyl-L-lysine) is present in human plasma, saliva, and urine but declines with age. It is proposed that GHK functions as a complex with copper 2+ which accelerates wound healing and skin repair. GHK stimulates both synthesis and breakdown of collagen and glycosaminoglycans and modulates the activity of both metalloproteinases and their inhibitors. It stimulates collagen, dermatan sulfate, chondroitin sulfate, and the small proteoglycan, decorin. It also restores replicative vitality to fibroblasts after radiation therapy. The molecule attracts immune and endothelial cells to the site of an injury. It accelerates wound-healing of the skin, hair follicles, gastrointestinal tract, boney tissue, and foot pads of dogs. It also induces systemic wound healing in rats, mice, and pigs. In cosmetic products, it has been found to tighten loose skin and improve elasticity, skin density, and firmness, reduce fine lines and wrinkles, reduce photodamage, and hyperpigmentation, and increase keratinocyte proliferation. GHK has been proposed as a therapeutic agent for skin inflammation, chronic obstructive pulmonary disease, and metastatic colon cancer. It is capable of up- and downregulating at least 4,000 human genes, essentially resetting DNA to a healthier state. The present review revisits GHK's role in skin regeneration in the light of recent discoveries.

## 1. Introduction

GHK is a tripeptide with the amino acid sequence glycyl-histidyl-lysine. It naturally occurs in human plasma, saliva, and urine. In plasma the level of GHK is about 200 ng/mL (10^−7^ M) at age 20, but declines to 80 ng/mL by age 60. This decline in the GHK-level coincides with the noticeable decrease in regenerative capacity of an organism. The human peptide GHK-Cu was isolated in 1973 by Pickart as an activity in human albumin that caused old human liver tissue to synthesize proteins like younger tissue [1]. Subsequent studies established this activity as a tripeptide with an amino acid sequence glycyl-L-histidyl-L-lysine with a strong affinity for copper that readily formed the complex GHK-Cu. It was proposed that GHK-Cu functions as a complex with copper 2+ [2]. Pickart et al. have established that GHK-Cu accelerates wound healing and contraction, improves the take of transplanted skin, and also possesses antiinflammatory actions [3–5].

Subsequent studies directed by Borel and Maquart et al. demonstrated that GHK-Cu at a very low, nontoxic concentration (1–10 nanomolar) stimulated both synthesis and breakdown of collagen and glycosaminoglycans [6]. GHK modulated an activity of both metalloproteinases and their inhibitors (TIMP-1 and TIMP-2), acting as a main regulator of wound healing and skin remodeling processes [7, 8]. GHK-Cu stimulated collagen, dermatan sulfate, chondroitin sulfate, and a small proteoglycan, decorin [9]. In 2001 McCormack et al. established that GHK-Cu restored replicative vitality to fibroblasts from patients after anticancer radiation therapy that damages cellular DNA [10]. GHK was also found to attract immune and endothelial cells to the site of an injury [11].

Wound healing activity of GHK-Cu was confirmed in animal experiments. GHK-Cu accelerated wound healing and increased blood vessel formation and the level of antioxidant enzymes in rabbits. This molecule also induced systemic wound healing in rats, mice, and pigs. It improved the healing of diabetic and ischemic wounds in rats, decreasing the level of TNF-alpha and stimulating collagen synthesis. It also facilitated healing of pad wounds in dogs [12–17]. Such well-documented skin regeneration activity prompted widespread use of GHK in antiaging cosmetic products [18].

Recently, GHK-Cu has been gaining publicity as a prospective therapeutic agent for chronic obstructive pulmonary disease (COPD), skin inflammation, and metastatic colon cancer [19–21]. It has been established that it is capable of up- and downregulating at least 4,000 genes in the human genome, essentially resetting DNA back to a healthier state [22]. These studies shed new light on the skin remodeling activity of the GHK-Cu peptide.

The present review revisits GHK-Cu's role in skin regeneration in the light of recent discoveries.

## 2. GHK Restores TGF-Beta Pathway in COPD Lungs

A collaborative study conducted by scientists from Boston University, University of Groningen, University of British Columbia, and University of Pennsylvania established that the GHK peptide reverses the gene expression signature of COPD, which is manifested by emphysema, inflammation, lung tissue destruction, and significant reduction of lung capacity.

The researchers identified 127 genes whose expression was significantly associated with emphysema severity. Among those genes, whose expression was upregulated in COPD patients, were genes involved in inflammation, while expression of genes involved in tissue remodeling and repair was markedly downregulated. Among the genes displaying decreased activity were genes involved in the TGF-beta pathway. Using the Connectivity Map, a software gene profiling tool developed by the Broad Institute, the researchers identified GHK as a compound which reversed changes in gene expression associated with emphysematous destruction. In particular, patients with COPD displayed a decreased activity of genes involved in the TGF-beta pathway. GHK reversed the gene expression pattern so it became consistent with the activation of the TGF-beta pathway.

*In vitro* studies confirmed that treating lung fibroblasts with GHK reversed negative changes associated with decreased TGF-beta activity. It has been established that lung fibroblasts derived from COPD patients had certain defects, which impaired their ability to contract and remodel collagen gel. When such fibroblasts were treated with either GHK or TGF-beta, the contraction and remodeling of collagen gel was restored and became comparable to fibroblasts derived from lungs of exsmokers without COPD. After GHK treatment, the lung fibroblasts derived from COPD patients were able to remodel collagen gel into fibrils. They also had an elevated expression of integrin beta 1. These findings indicate that GHK may be able to improve tissue regeneration by restoring activity of genes involved in the TGF-beta pathway [23].

It is known that skin regeneration requires the participation of multiple cytokines and growth factors. Rather than work separately, they engage in a crosstalk, which involves interaction of different cellular pathways. For example, cellular pathways regulated by TGF-beta and integrins seem to be connected [24]. GHK's ability to restore the contraction and remodeling of collagen gel in the COPD study demonstrate that GHK is capable of activating both TGF-beta and integrin beta 1 pathways during tissue regeneration. Even though the exact mechanism of GHK's action is yet to be elucidated, it becomes apparent that the diverse and multiple effects of GHK in skin regeneration can be better understood through its ability to reset the gene pattern back to a healthier state, thereby leading to the activation or deactivation of various cellular pathways.

## 3. Cancer Metastasis Genes and Skin Remodeling

Hong et al. used genome-wide profiling to identify genetic biomarkers for metastasis prone colorectal cancer as well as their perturbagens, substances that modulated their expression. The search out of 1309 bioactive compounds yielded only two substances that were able to effectively downregulate expression of “metastatic” genes, GHK and the plant alkaloid, securinine. GHK suppressed RNA production in 70% of 54 human genes overexpressed in patients with an aggressive metastatic form of colon cancer. GHK produced the result at a low non-toxic 1 micromolar concentration and securinine at 18 micromolar. The authors point out that both GHK and securinine are well-known skin remodeling agents. Securinine activates macrophages and is a component of traditional African and Chinese medicines for skin injuries [25]. The 54 genes whose expression was reversed by GHK included “node molecules” YWHAB, MAP3K5, LMNA, APP, GNAQ, F3, NFATC2, and TGM2, all of which are involved in regulation of multiple biological functions through a complex molecular network [20]. The fact that GHK was able to suppress 70% of genes involved in the development of an aggressive metastatic form of colon cancer indicates that GHK is capable of the regulation of various biochemical pathways on a gene level and it seems to be resetting the gene activity back to health, which leads to the improvement of tissue repair.

*In vitro* studies by Matalka et al. found that when three lines of human cancer cells (SH-SY5Y neuroblastoma cells, U937 histolytic cells, breast cancer cells) were incubated in culture with 1 to 10 nanomolar GHK, the programmed cell death system (apoptosis) was reactivated and cell growth inhibited [26].

Pickart et al., using the Broad Institute data, found that GHK induces anti-cancer expression of numerous caspase, growth regulatory, and DNA repair genes. The combination of ascorbic acid and GHK-Cu strongly inhibited the growth of sarcoma-180 in mice [27].

## 4. Recovery of Skin Stem Cells

Skin regeneration depends on viability and proliferative potential of stem cells. Skin proliferation starts in the basal layer of keratinocytes, which are attached to the basal membrane. When a cell leaves the basal layer, it undergoes terminal differentiation. Stem cells have unlimited self-renewal capacity; however, their proliferative potential declines with age. GHK-Cu, in concentrations of 0.1–10 micromolar, increased expression of epidermal stem cell markers such as integrins and p63 in basal keratinocytes in dermal skin equivalents, which according to the authors indicate increased stemness and proliferative potential of basal keratinocytes. Therefore, restoration of gene pattern characteristic of healthy stem cells, which leads to activation of integrins and p63 cellular pathways may be another target of GHK's gene modulatory activity relevant to skin regeneration [28, 29].

A recent study has established that pretreatment of human mesenchymal stem/stromal cells (MSC) with GHK presented in a biodegradable carrier (alginate gel) produced a dose-dependent increase in secretion of proangiogenic factors such as the vascular endothelial growth factor (VEGF) and basic fibroblast growth factor. When pretreated with antibodies to integrins alpha 1 and beta 1, MSC failed to produce an increase of VEGF, which indicated that the effects of GHK on secretion of trophic factors by MSC involve the integrin cellular pathway [30].

## 5. GHK and IL-6 in Skin Repair

Wound healing and skin repair involves inflammation, cell proliferation and migration and dermal matrix remodeling. Excessive inflammation may delay healing and lead to scar formation. The copper complexes of the peptides Gly-Gly-His (GGH), Gly-His-Lys (GHK) reduced TNF-alpha induced secretion of proinflammatory cytokine IL-6, in normal human dermal fibroblasts, while saccharomyces/copper ferment (OligolidesA Copper) had no effect. The authors propose that GHK and GHK-Cu can be used as a topical agent in treatment of inflammatory skin conditions instead of corticosteroids [21].

## 6. GHK and DNA Repair

GHK was able to restore viability of irradiated fibroblasts. The researchers used cultured human fibroblasts obtained from cervical skin that was either intact or exposed to radioactive treatment (5000 rad). GHK (10^−9^ M) was added in a serum free medium directly to the cell culture. An equivalent amount of plain serum-free medium was added to control cells. Although irradiated fibroblasts survived and replicated in the cell culture, their growth dynamics were markedly different from that of intact cells. The growth of the irradiated cells was especially delayed at 24 and 48 hour measurements. However, the irradiated fibroblasts treated with GHK showed much faster growth that was similar to the normal (non-irradiated control cells). In addition, GHK-treated irradiated fibroblasts showed higher production of growth factors, which are essential for wound healing [31].

Fibroblasts are central cells in both wound healing and tissue renewal processes. They not only synthesize different components of dermal matrix, but also produce a number of growth factors that are involved in a multitude of cellular pathways regulating cell migration and proliferation, angiogenesis, epithelialization, and so forth. Radiation damages cell DNA, thus impairing their function. Since GHK was able to restore function of irradiated fibroblasts, it has to have effects on DNA repair.

Studies using the Broad Institute's Connectivity map found that GHK significantly increased the expression of DNA repair genes with 47 genes stimulated and 5 genes suppressed (more than or equal to a 50% increase or decrease) [22].

## 7. Facial Studies

A number of placebo-controlled clinical studies found GHK-Cu to improve skin quality in women around the age of 50. A study of collagen production determined by studying skin biopsy samples using immunohistological techniques found that after applying creams to the thighs for one month, GHK-peptides had a significant effect on collagen production. Increases were found in 70% of the women treated with GHK-Cu, in contrast to 50% treated with the vitamin C cream, and 40% treated with retinoic acid [32].

A GHK-Cu facial cream reduced visible signs of aging after 12 weeks of application to the facial skin of 71 women with mild to advanced signs of photoaging. The cream improved skin laxity, clarity, and appearance, reduced fine lines and the depth of wrinkles, and increased skin density and thickness [33].

A GHK-Cu eye cream, tested on 41 women for twelve weeks with mild to advanced photodamage, was compared to a placebo control and an eye cream containing vitamin K. The GHK-Cu cream performed better than both controls in terms of reducing lines and wrinkles, improving overall appearance, and increasing skin density and thickness [34].

In another 12-week facial study of 67 women between 50 and 59 years with mild to advanced photodamage, a GHK-Cu cream was applied twice daily and improved skin laxity, clarity, firmness and appearance, reduced fine lines, coarse wrinkles and mottled pigmentation, and increased skin density, and thickness. The cream also strongly stimulated dermal keratinocyte proliferation as determined by the histological analysis of biopsies [35].

These placebo-controlled studies demonstrated that GHK-Cu skin creams had the following effects:

1. Tighten loose skin and improve elasticity.
2. Improve skin density and firmness.
3. Reduce fine lines and deep wrinkles.
4. Improve skin clarity.
5. Reduce photodamage and mottled hyper-pigmentation.
6. Strongly increase keratinocyte proliferation.

## 8. Formulation and Delivery

GHK-Cu appears to pass the skin's horny layer (stratum corneum) in quantities sufficient to activate regenerative events. The permeability coefficients of copper complexes increase with increasing pH. It was proved that only the tripeptide GHK and its complexes with copper: GHK-Cu and (GHK)(2)-Cu are able to migrate through the membrane model of the stratum corneum [36–38]. Yet, because of its susceptibility to the actions of proteolytic enzymes it is important to ensure its sustained delivery in bioactive concentrations. Arul et al. proposed the use of biotinylated peptide incorporated collagen matrix (Boc-GHK) for dermal wound healing. They observed improved wound contraction and increased cell proliferation and a high expression of antioxidant enzymes in wounds treated with Boc-GHK compared to the control [39].

A recent study investigated formulation requirements for GHK. It has been established that the peptide was prone to hydrolytic cleavage when subjected to oxidative stressors. It was stable in water in the pH range 4.5–7.4 buffers for at least two weeks at 60°C. The distribution coefficients in octanol-phosphate buffered saline indicated the highly hydrophilic nature of GHK-Cu with log *D* values between −2.38 and −2.49 at a pH range of 4.5–7.4. GHK-Cu can be incorporated into Span 60 based niosomes. It is less stable in the presence of the negatively charged lipid diacetyl phosphate [40].

Glycyl-L-histidyl-L-lysine-Cu(II) (GHK-Cu(2+))-loaded Zn-pectinate microparticles in the form of hydroxypropyl cellulose (HPC) compression-coated tablets were developed for colon delivery of GHK. The release of GHK-Cu(2+) from Zn-pectinate microparticles was strongly affected by the cross-linking agent concentration and drug amount, but not by surfactant amount. The microparticles released 50–80% of their drug load within 4 hours. The optimum microparticle formulation (F8) coated with a relatively hydrophobic polymer HPC presented a colonic delivery system. This study indicates a possibility of including GHK into a delivery system for internal use. It should be possible to incorporate GHK into a dietary supplement with many health promoting properties and no side effects. Such formulations can be used to improve dermal healing in addition to topical delivery [41].

## 9. Breakdown Resistant Mixed Copper Peptide Complexes

A major problem in the treatment of human skin wounds and ulcers is dealing with infected wounds. The powerful bacteria in such wounds secrete proteases that can rapidly breakdown GHK and other types of healing growth factors.

GHK itself is formed during protein breakdown in the company of a large number of other small peptides. When we added copper 2+ to the entire mixture of small peptides formed during breakdown, we found that such a mixture had significant wound healing activity. Moreover, such peptides were resistant to further breakdown. The details of preparing these mixed copper peptide complexes and their incorporation into wound healing creams are given in the referenced US patents [42, 43].

Howard Maibach's group later tested the mixed copper peptide complexes using four human wound healing systems.

These skin damaging methods were

1. removal of skin lipids with acetone,
2. irritation of skin with strong sodium lauryl sulfate,
3. irritation of skin by tape stripping,
4. activation of an allergic response in patients with nickel allergies.

In all four studies, there was a more rapid healing with creams containing the mixed copper peptide complexes than with the control creams without the complexes. There was also a more rapid reduction in the erythema (redness) in the nickel allergy patients [44–47].

## 10. Biochemistry of GHK-Cu

**Figure 1:**
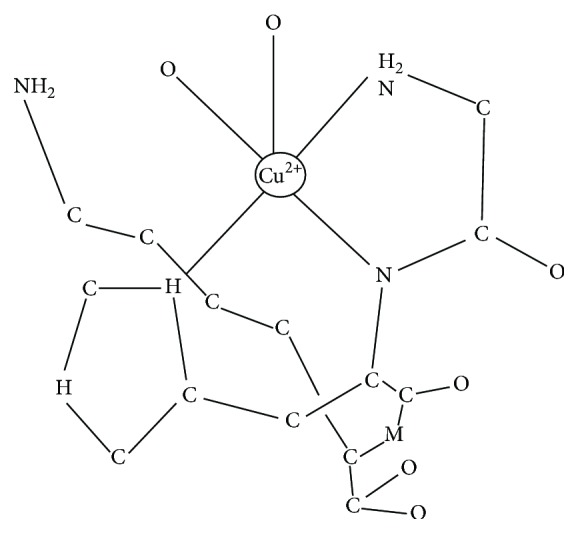
Molecular crystal structure of the tripeptide GHK-Cu. In solution, lysine carboxyl groups of neighboring complexes may participate in a complex formation.

The molecular structure of the GHK copper complex (GHK-Cu) has been extensively studied using X-ray crystallography, EPR spectroscopy, X-ray absorption spectroscopy, and PMR spectroscopy as well as other methods such as titration. In the GHK-Cu complex, the Cu (II) ion is coordinated by the nitrogen from the imidazole side chain of the histidine, another nitrogen from the alpha-amino group of glycine, and the deprotonated amide nitrogen of the glycine-histidine peptide bond (Figure 1).

Lau and Sarkar found that at the physiological pH, GHK-Cu complexes can form binary and ternary structures which may involve the amino acid histidine and/or the copper binding region of the albumin molecule. They also observed that GHK can easily obtain copper 2+ bound to the high affinity copper transport site on plasma albumin (albumin's binding constant of log_10_ = 16.2 versus GHK's binding constant of log_10_ = 16.44). It has been established that the copper (II) redox activity is silenced when copper ions that are bound to the GHK tripeptide, which allows the delivery of nontoxic copper into the cell [48–50].

The most distinctive feature of GHK is its ability to form complexes with copper (II) [51]. This is very important since copper is required for more than a dozen vital enzymes in the human body and skin including those that participate in connective tissue formation, antioxidant defense, and cellular respiration. Copper also exhibits signaling function and can influence cell behavior and metabolism. For example, sufficient copper is required for stem cells to start proliferating and repairing tissues. GHK also helps to reduce the level of free ionic copper thus preventing the possibility of oxidative damage.

Apart from being able to bind with copper, GHK can also quench some toxins, in particular those that are generated during lipid peroxidation [52]. This makes GHK a quite efficient antioxidant.

Finally, GHK has been shown to be able to serve as a cell adhesion molecule, which means that it helps cells to attach themselves to the extracellular matrix. This facilitates migration, proliferation, and differentiation of repair cells in the skin [53, 54].

## 11. GHK: A Built-In Natural Regulator of Dermal Repair

The wound healing process in the skin goes through the following phases: hemostasis (blood clotting), inflammation, granulation, and scar remodeling. Every stage requires well-coordinated cell interaction and therefore is precisely orchestrated by a plethora of biological active molecules coming from different sources.

For example, immediately after injury degranulating platelets release growth factors (such as TGF-beta) that mobilize immune cells and attract them to the site of the injury.

Keratinocytes and fibroblasts also produce a multitude of growth factors. Neutrophils, macrophages, and other immune cells that get recruited to the site of injury produce their share of growth factors and cytokines, as well.

GHK is a rare human sequence in proteins; however, it is more common in proteins of the extracellular matrix (ECM). GHK triplet is present in the alpha 2(I) chain of type I collagen and can be liberated by proteases at the site of a wound [55]. One of the most notable GHK-containing proteins that is always present in sites of remodeling is glycoprotein SPARC. Its proteolytic breakdown after injury generates GHK-Cu [56]. It has long been established that proteolytic breakdown of some proteins and proteoglycans of ECM results in the release of important regulatory molecules, matrikines [57]. These molecules activate and regulate dermal repair processes. The ability of GHK to reset the genome to a healthier gene pattern which leads to a better regulation of various cellular pathways can explain its diverse dermal repair actions. Since GHK is present as an amino acid sequence in proteins of ECM and is released after an injury, it appears to serve as a natural built-in modulator of dermal repair.

## 12. Conclusion

It has long been accepted that the human copper-binding peptide GHK-Cu enhances healing of dermal wounds and stimulates skin renewal exhibiting a wide range of effects. Cellular pathways involved in dermal repair and skin regeneration form an intricate and finely orchestrated biochemical network, where various regulatory molecules are involved in a cross-talk. When such an interaction is disrupted, the healing is delayed and may result in excessive inflammation and scarring. It appears that GHK is able to restore healthy functioning of essential cellular pathways in dermal repair through resetting the gene pattern to a healthier state.

GHK, abundantly available at low cost in bulk quantities, is a potential treatment for a variety of disease conditions. The molecule is very safe and no issues have ever arisen during its use as a skin cosmetic or in human wound healing studies.

Based on our studies where GHK was injected in a distant part of a body, such as thigh to induce systemic healing, and also on studies where GHK was injected intraperitoneally once daily to induce systemic wound healing throughout the body, we estimate that about 100–200 mgs of GHK will produce therapeutic actions in humans. But even this may overestimate the necessary effective dosage of the molecule [14].

Studies where GHK was used for the healing of bone fractures in rats used a mixture of small molecules (Gly-His-Lys (0.5 *μ*g/kg), dalargin (1.2 *μ*g/kg) (an opioid-like synthetic drug), and the biological peptide thymogen (0.5 *μ*g/kg) (L-glutamyl-L-tryptophan)) to heal bones. The total peptide dosage is about 2.2 micrograms per kilogram or if scaled for the human body, about 140 micrograms per injection with treatments for 10 days [58, 59]. GHK can be incorporated into topical gels, used in dermal patches, and collagen membranes, as well as being administered orally in liposomes and other carriers. Future research is needed to establish the effective dosage in humans and the best ways of delivery.
